# Supplementary material for: BLTP3A is associated with membranes of the late endocytic pathway and is an effector of CASM
Source: EMBO J. 2025 Sep 11;44(21):6168–95. doi: 10.1038/s44318-025-00543-9 (PMC12583604; doi:10.1038/s44318-025-00543-9)
Supplement: Supplementary file 10 — Movie EV7 [file 44318_2025_543_MOESM10_ESM.zip › Movie_EV7_legend.rtf]

Movie EV7Time lapse fluorescence imaging of RPE-1 cells expressing BLTP3A∆IR-RFP and mApple-IST1 treated with 1 mM LLOMe after the first frame. Time, 900 s. Interval, 10 sec. Scale bar, 5 µm.
