# Supplementary material for: BLTP3A is associated with membranes of the late endocytic pathway and is an effector of CASM
Source: EMBO J. 2025 Sep 11;44(21):6168–95. doi: 10.1038/s44318-025-00543-9 (PMC12583604; doi:10.1038/s44318-025-00543-9)
Supplement: Supplementary file 11 — Movie EV8 [file 44318_2025_543_MOESM11_ESM.zip › Movie_EV8_legend.rtf]

Movie EV8FIB-SEM reconstruction of GFP-LC3B and BLTP3A-mRFP-positive lysosomes in an RPE-1 cell 15 min after LLOMe addition (from Figure 5E). Reconstructed organelle colors: lysosome, dark green; ER, yellow; mitochondria, blue; small vesicles, magenta; large vesicles, green. Scale bar, 1 μ. 
