# Supplementary material for: BLTP3A is associated with membranes of the late endocytic pathway and is an effector of CASM
Source: EMBO J. 2025 Sep 11;44(21):6168–95. doi: 10.1038/s44318-025-00543-9 (PMC12583604; doi:10.1038/s44318-025-00543-9)
Supplement: Supplementary file 12 — Movie EV9 [file 44318_2025_543_MOESM12_ESM.zip › Movie_EV9_legend.rtf]

Movie EV9FIB-SEM image stack of the region used for the reconstruction in Figure 5E and EV Movie 5. Lysosomes are pseudo-colored dark green. Scale bar, 1 μ. 
