# Supplementary material for: BLTP3A is associated with membranes of the late endocytic pathway and is an effector of CASM
Source: EMBO J. 2025 Sep 11;44(21):6168–95. doi: 10.1038/s44318-025-00543-9 (PMC12583604; doi:10.1038/s44318-025-00543-9)
Supplement: Supplementary file 13 — Movie EV10 [file 44318_2025_543_MOESM13_ESM.zip › Movie_EV10_legend.rtf]

Movie EV10Time lapse fluorescence imaging of RPE-1 cells expressing BLTP3A-RFP and GFP-LC3B treated with MSU crystals for 2 hrs. Time, 1500 s. Interval, 30 sec. Scale bar, 2 µm.
