# Supplementary material for: BLTP3A is associated with membranes of the late endocytic pathway and is an effector of CASM
Source: EMBO J. 2025 Sep 11;44(21):6168–95. doi: 10.1038/s44318-025-00543-9 (PMC12583604; doi:10.1038/s44318-025-00543-9)
Supplement: Supplementary file 14 — Expanded View Figures [file 44318_2025_543_MOESM14_ESM.pdf]

Expanded View Figures

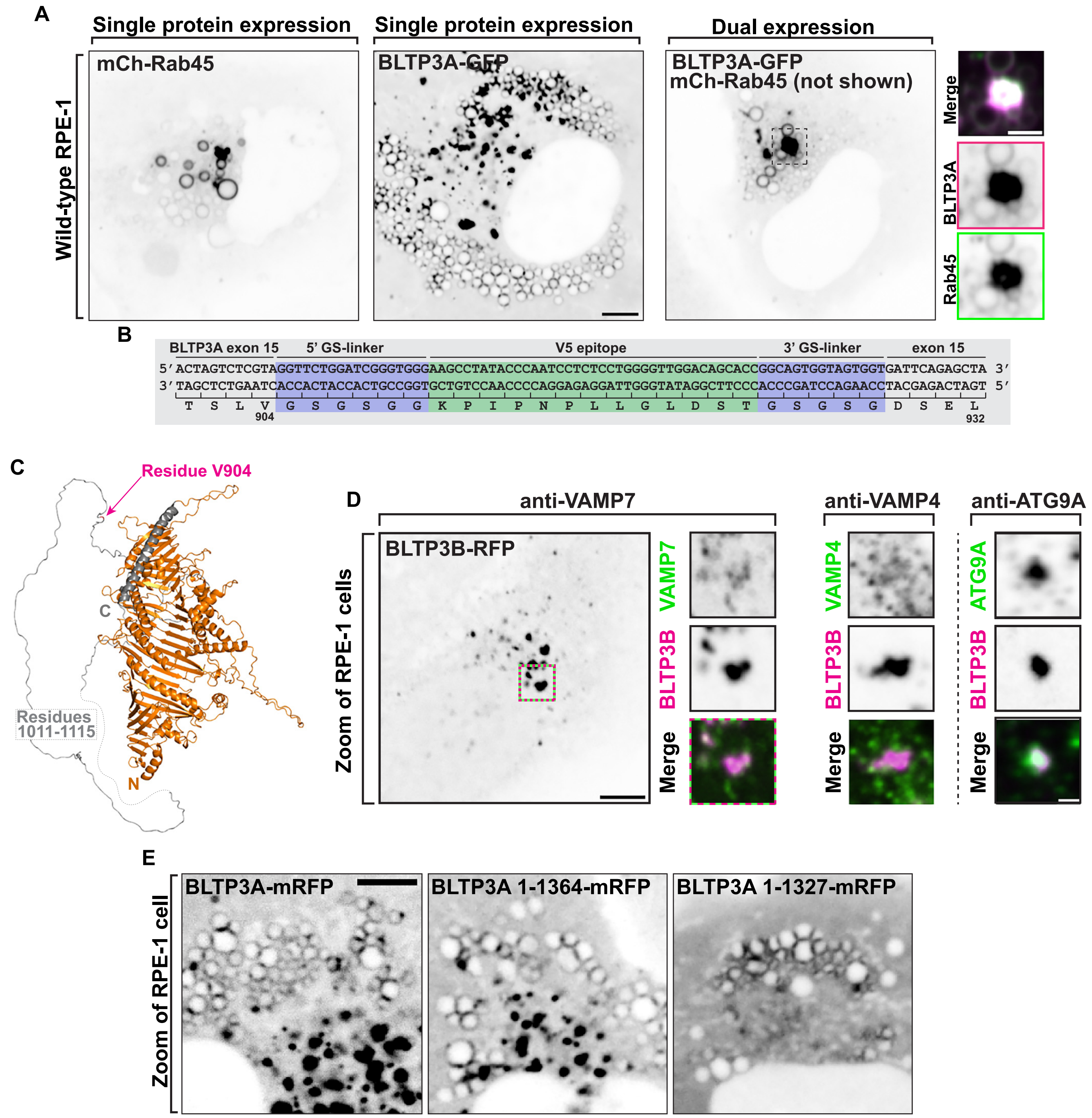

Figure EV1. BLTP3A localizes with endo-lysosomal proteins.

(**A**) Live fluorescence images (inverted grays) of RPE-1 cells expressing either GFP-Rab45 (left), BLTP3A-mRFP (center), or both proteins together (only BLTP3A is shown) (right) as indicated. Scale bar, 5 μm. High-magnification scale bar, 2 μm. (**B**) Genomic sequence of the edited BLTP3A locus (insertion of the V5 epitope) in A549 cell. Blue, small Gly-Ser linkers; green, V5 epitope sequence. (**C**) AlphaFold prediction of BLTP3A. The site where the V5 epitope (V904) was inserted is indicated. The long disordered sequence and the C-terminal helix are shown in gray. (**D**) Left: Fluorescence image of an RPE-1 cell expressing exogenous BLTP3B-mRFP (inverted grays) and immunolabeled with antibodies against endogenous VAMP7 (shown at right in the high magnification of the squared region in the main field). Scale bar, 5 μm. Right: zooms of different RPE-1 cells expressing exogenous BLTP3B-mRFP (magenta) and immunolabeled with antibodies (green) against endogenous VAMP4 or ATG9A. Individual channels are shown as inverted grays. Merge of channels on bottom. Scale bar, 1 μm. (**E**) Fluorescence images of RPE-1 cells expressing the indicated BLTP3A-mRFP construct. Scale bar, 5 μm.

 © The Author(s)

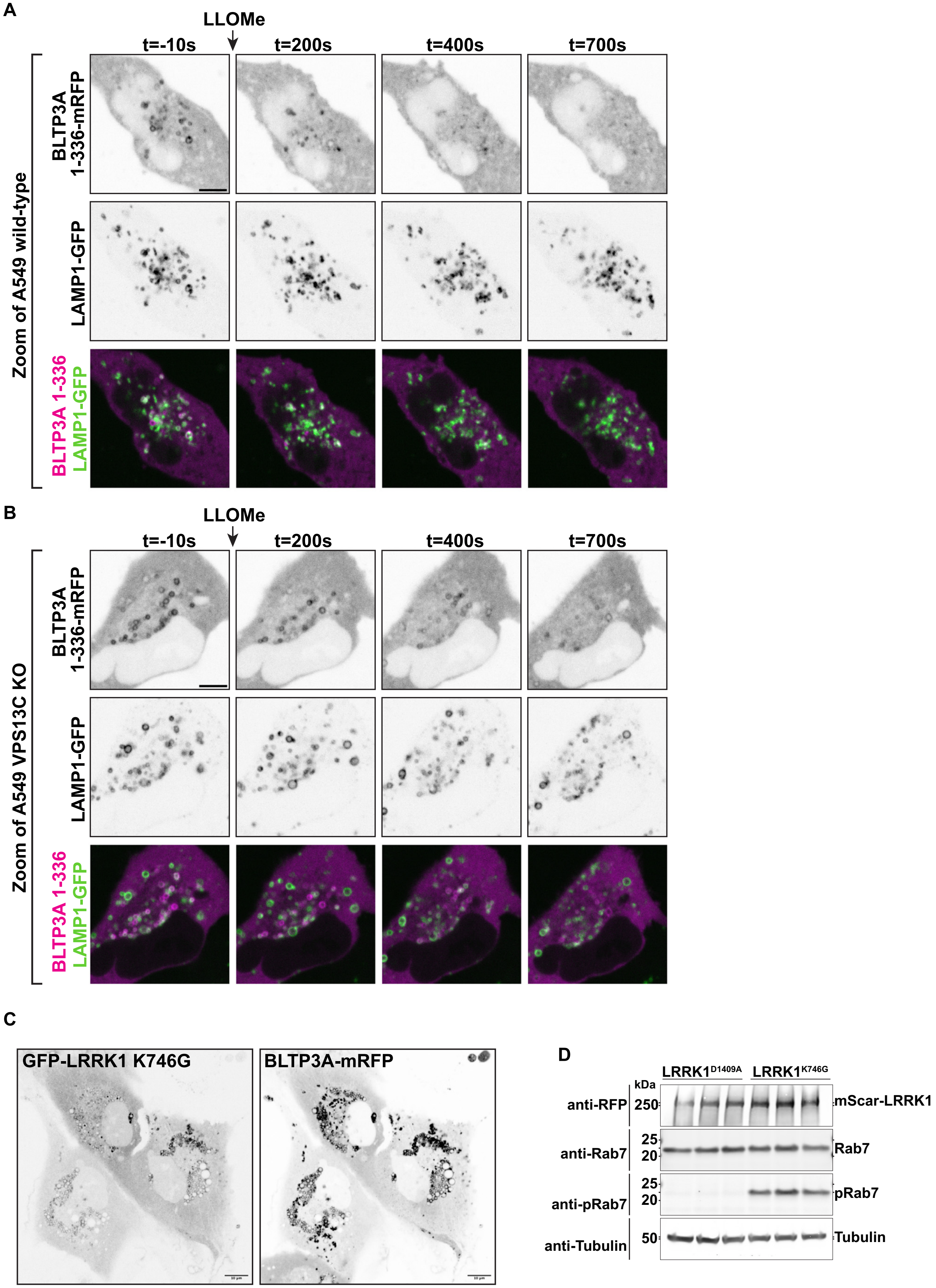
A
LLOMe
t=-10s
t=200s
t=400s
t=700s
Zoom of A549 wild-type
BLTP3A 1-336-mRFP
LAMP1-GFP
BLTP3A 1-336
LAMP1-GFP
B
LLOMe
t=-10s
t=200s
t=400s
t=700s
Zoom of A549 VPS13C KO
BLTP3A 1-336-mRFP
LAMP1-GFP
BLTP3A 1-336
LAMP1-GFP
C
GFP-LRRK1 K746G
BLTP3A-mRFP
D
LRRK1 D1409A
LRRK1 K746G
kDa
anti-RFP
250
mScar-LRRK1
anti-Rab7
25
20
Rab7
anti-pRab7
25
20
pRab7
anti-Tubulin
50
Tubulin

Figure EV2. BLTP3A is displaced from the surface of lysosomes upon lysosomal damage.

(**A**) Time-series of live fluorescence images of BLTP3A-1-336-mRFP (magenta) and the lysosomal marker LAMP1-GFP (green) expressed in wild-type A549 cells before and after addition of LLOMe. Fluorescence of individual channels is shown in inverted grays. Scale bar, 5 µm. (**B**) Time-series of live fluorescence images of BLTP3A-1-336-mRFP (magenta) and the lysosomal marker LAMP1-GFP (green) expressed in VPS13C KO A549 cells before and after addition of LLOMe. Fluorescence of individual channels is shown in inverted grays. Scale bar, 5 µm. (**C**) Live fluorescence images (inverted grays) of RPE-1 cells expressing exogenous GFP-LRRK1^{K746G} (left) and BLTP3A-mRFP (right). A partial association of BLTP3A-mRFP and GFP-LRRK1^{K746G} was observed. Scale bar, 10 µm. (**D**) Western blot of lysate of RPE-1 cells expressing exogenous RFP-LRRK1^{K746G} or RFP-LRRK1^{D1409A} for RFP (to detect LRRK1 fusions), Rab7, phospho-Rab7 S72, and alpha-tubulin as a loading control. Individual lanes are biological replicates.

 © The Author(s)

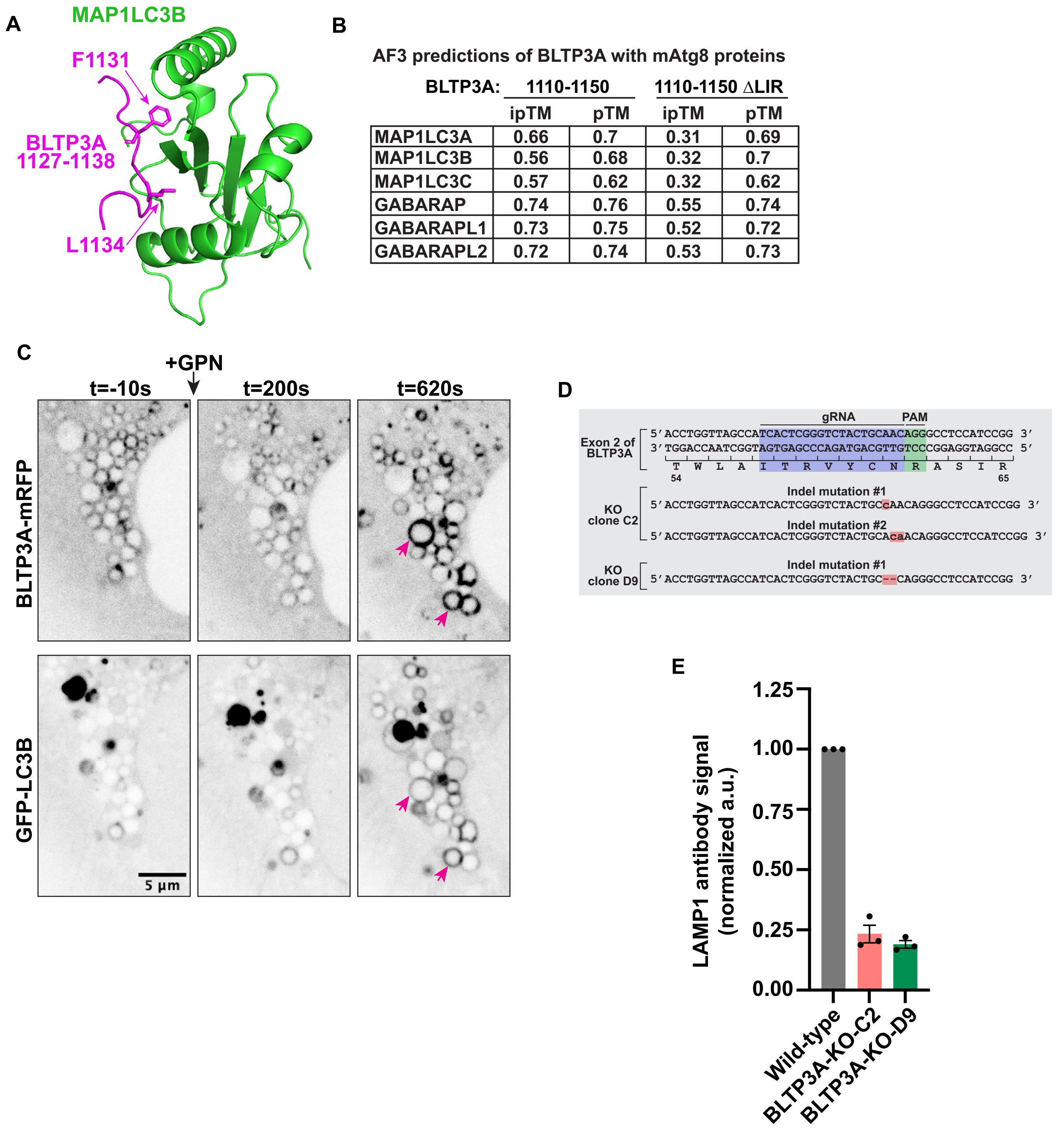

BLTP3A:	1110-1150		1110-1150 ΔLIR	
	ipTM	pTM	ipTM	pTM
MAP1LC3A	0.66	0.7	0.31	0.69
MAP1LC3B	0.56	0.68	0.32	0.7
MAP1LC3C	0.57	0.62	0.32	0.62
GABARAP	0.74	0.76	0.55	0.74
GABARAPL1	0.73	0.75	0.52	0.72
GABARAPL2	0.72	0.74	0.53	0.73

Figure EV3. BLTP3A is an effector of CASM.

(**A**) AlphaFold3 multimer prediction of full-length MAP1LC3B (green) and a.a. 1110–1150 of BLTP3A (magenta). Arrows indicate key residues of the LIR motif of BLTP3A. (**B**) AlphaFold3 multimer predictions of mATG8 proteins and a.a. 1110–1150 of BLTP3A with and without the LIR motif (ΔLIR). (**C**) Time-series of live fluorescence images (inverted grays) of BLTP3A-mRFP and GFP-LC3B before and after addition of GPN. Arrowheads point to lysosomes where BLTP3A and LC3B decorate the entire profile upon addition of GPN. Time, seconds. Scale bar, 5 μm. (**D**) Genomic sequence of the edited BLTP3A locus in A549 cell. Blue, gRNA; green, PAM; red, indel mutations. (**E**) Quantification of relative LAMP1 expression from western blots ($N = 3$, biological replicates) of Fig. 7A. Error bars indicate the standard error of the mean (SEM).

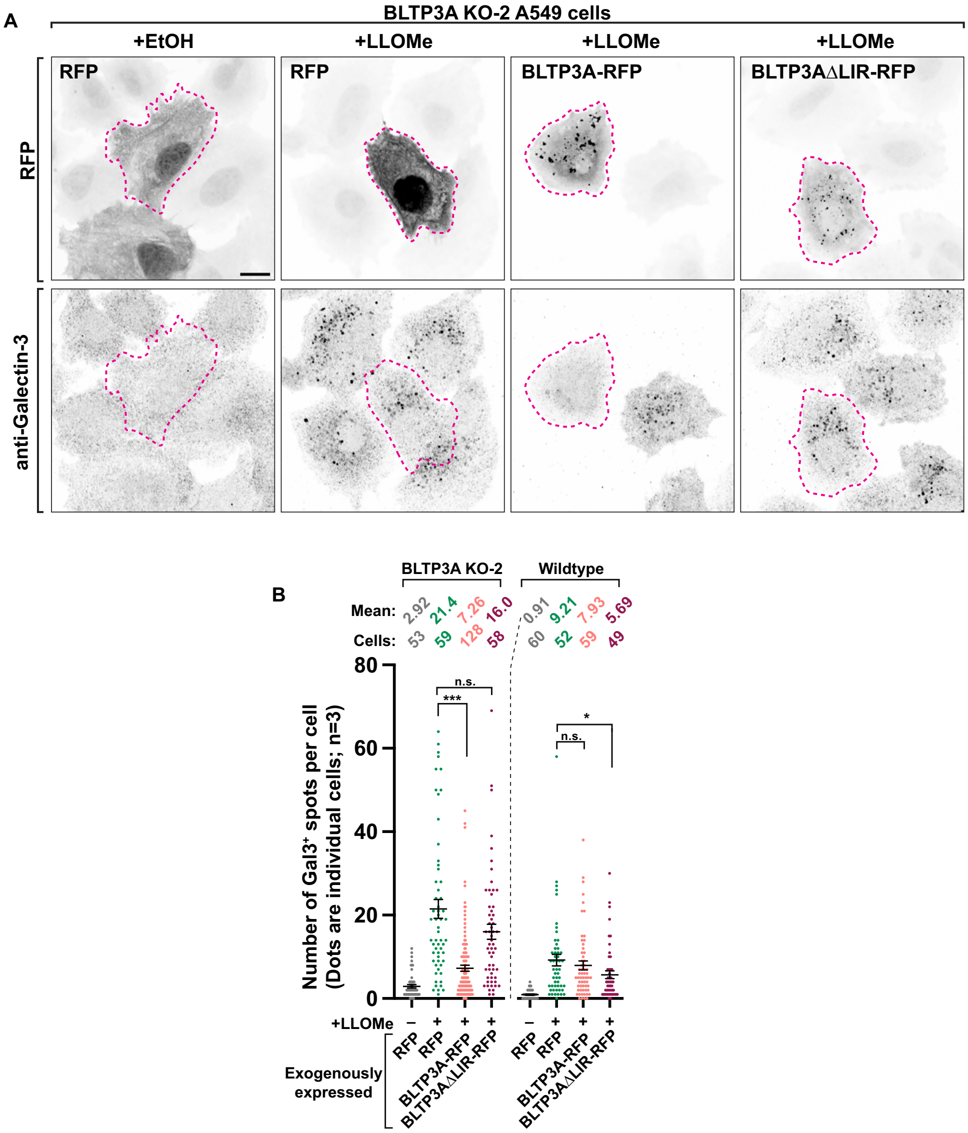

Figure EV4. Lysosomal fragility from loss of BLTP3A is rescued by BLTP3A over-expression in A549 cells.

(**A**) Fluorescence images (inverted greys) of BLTP3A KO A549 cells expressing indicated RFP protein (top row) with antibodies against galectin-3 (bottom row). Dotted magenta line indicates cell boundary. Cells were treated with vehicle control (left column) or 1 mM LLOMe (right three columns). Scale bar, 10 μm. (**B**) Quantification of galectin-3 spots per cell from field (**A**) ($N = 3$, biological replicates.). Error bars report the standard error of the mean (SEM). $^{***}P < 0.001$; $^{**}P < 0.01$; $^{*}P < 0.05$; n.s., not significant. Mean number of galectin-3 spots per cell and number of cells counted per condition indicated.

 © The Author(s)
